# Intratubular *Enterococcus faecalis* viability assessment following root canal instrumentation with rotary and reciprocating systems via fluorescence microscopy

**DOI:** 10.34172/joddd.2020.044

**Published:** 2020-12-02

**Authors:** Jamileh Ghoddusi, Ehsan Arian, Maryam Golmohammadi, Maryam Gharechahi, Siavash Moushekhian

**Affiliations:** ^1^Dental Research Center, Faculty of Dentistry, Mashhad University of Medical Sciences (MUMS), Mashhad, Iran; ^2^Antimicrobial Resistance Research Center, Mashhad University of Medical Sciences, Mashhad, Iran; ^3^Department of Endodontics, Khorasan Shomali University of Medical Sciences, Bojnord, Iran; ^4^Dental Materials Research Center, Faculty of Dentistry, Mashhad University of Medical Sciences (MUMS), Mashhad, Iran

**Keywords:** Antimicrobial test, Bacterial viability, *Enterococcus faecalis*, Instrumentation, ProTaper Gold system, Root canal preparation, WaveOne systems

## Abstract

**Background.** The present in vitro study aimed to compare the effectiveness of the WaveOne and ProTaper Gold systems in removing the *Enterococcus faecalis* biofilm.

**Methods.** Thirty-eight mandibular premolars were selected. The root canals were assigned to standard control (canals serially enlarged with ProTaper Gold S1-S2-F1-F2, n=15) and experimental (canals enlarged with Primary WaveOne file, n=15) groups. Following the instrumentation procedure, the root canals underwent a sampling procedure, and the colonyforming unit (CFU) counts were determined. The samples were also evaluated under a fluorescent microscope to evaluate viable bacteria. The data were analyzed using independent samples t test and paired samples *t* test.

**Results.** The results showed that, compared with the ProTaper group, the WaveOne group exhibited the least viable bacteria (*P* =0.004).

**Conclusion.** It was concluded that comparison with the ProTaper Gold rotary system, the WaveOne reciprocating file is more successful in reducing intratubular viable bacteria counts.

## Introduction

The principal etiologic factor for postoperative apical periodontitis is bacteria; therefore, root canal instrumentation is an important stage in root canal treatment.

Although chemomechanical procedures have provided significant bacterial reduction,^1^ no instrument or instrumentation technique can provide optimal disinfection of the root canal systems and make the root canal system free of bacteria.^2^

Modified instruments have been introduced to overcome the limitations and improve cleaning and disinfection procedures, one of which is the single-file system, which completely instruments the root canal with one single file.^3^ WaveOne (Dentsply Maillefer) system is one of M-wire reciprocating systems^4^ that increases instrument flexibility and its cyclic fatigue resistance.^5^ Reciprocating systems have demonstrated desirable effects in terms of cleaning and disinfecting abilities.^3,6^

This study aimed to compare the efficacy of the WaveOne reciprocating systems with the rotary system in eliminating intratubular *Enterococcus faecalis*biofilm using both culture and fluorescence microscopy methods.

## Methods

Thirty-eight extracted human mandibular premolars with a completely formed apex and a straight single root canal were selected for this study, disinfected with 3% sodium hypochlorite (NaOCl) for five minutes, and stored in distilled water until further use. All the teeth with two canals, isthmus, curvature, and obstruction on radiographs were excluded from the study.

**Figure 1:**
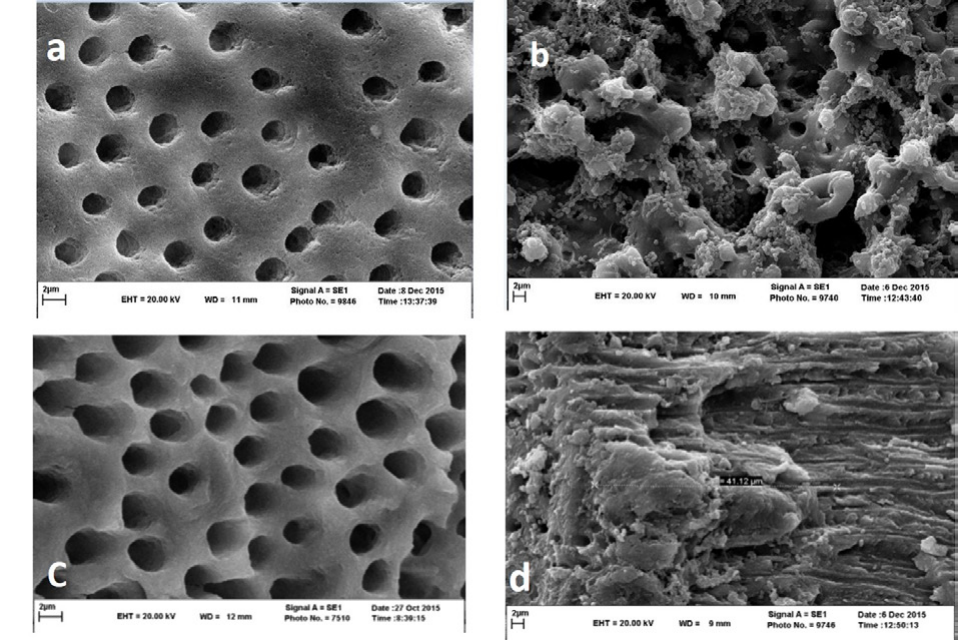
(a) SEM image of root canal walls; open dentinal tubules after smear layer removal and sterilization. (b) Colonization of bacteria on the root canal wall and in the dentinal tubules after bacterial contamination. (c) No bacterial growth in negative group during antimicrobial evaluation. (d) bacterial growth and dentinal penetration in positive control during antimicrobial evaluation.

The teeth were decoronated to standardize the root lengths to 13 mm. The patency of the canals was checked with a #10 K-file (Dentsply, Maillefer, Ballaigues, Switzerland). The coronal part of the canals was flared with #2 and #3 Gates-Glidden burs (Dentsply Maillefer, Ballaigues, Switzerland). Subsequently, 5 mL of 5.25% NaOCl was delivered by a syringe with a 27-gauge needle into each root canal. Finally, the smear layer was eliminated using 1 mL of 17% EDTA left in the root canal for one minute, followed by 5 mL of 5.25% NaOCl. Subsequently, 3 mL of saline solution was used as the final irrigation. The method’s efficiency was verified by negative controls (n=4) by SEM to observe the presence of open dentinal tubules (Figure 1a).

The root canals were dried with paper points, and two coats of nail varnish were applied over the root surfaces. Next, all the samples were sterilized in an autoclave at 121º and 15 Ibs of pressure for 20 minutes. Pure cultures of *E. faecalis.* (ATCC 29212), grown in a brain-heart infusion broth (BHI), were prepared to achieve the turbidity of a 2.0 McFarland standards [~6.0×10^8^ colony forming units (CFU)/mL]. The root canals (except for negative controls) were inoculated with an *E. Faecalis* suspension. The teeth were then incubated at 37ºC for 4 weeks. The medium was changed every two days to confirm *E. faecalis* growth.

SEM evaluation was used to confirm bacterial penetration into the dentinal tubules in the positive control group (n=4) (Figure 1b). Following incubation, the contaminated teeth were divided into two groups in terms of the instrumentation technique used as follows:

- Standard control group: The root canals were serially enlarged with ProTaper Gold S1-S2-F1-F2 (n=15), and irrigation was performed during the instrumentation with 5.25% NaOCl.
- Experimental group: The root canals were enlarged with the Primary WaveOne file (n=15), and irrigation was performed during the instrumentation with 5.25% NaOCl. The final irrigation was carried out using 5 mL of sterile saline solution, after which the root canals were dried with sterile paper paints.

To evaluate the efficiency of the instrumentation techniques, two antimicrobial tests were designed. Following the instrumentation procedures, dentinal segment samples were collected using the #3Gates-Glidden drill. Each bur was used three times in the entire length of the root canal. A new sterilized bur was used for each tooth. The dentinal shavings were transferred to tubes containing 500 μL of saline solution and vortexed and preserved for 1 minute. After 10-fold serial dilutions, aliquots of 50 μL were placed onto BHI agar plates, which were then incubated at 37ºC for 24 hours; the CFUs were numbered and based on the known dilution factors, they were transformed into actual counts.

For the second method, the rest of the suspensions in tubes were stained with fluorescent stains with LIVE/DEAD® BacLight^TM^ Viability kit (Molecular Probes Inc., Eugene, Oregon, USA), and centrifuged to concentrate; furthermore, their sediments were subjected to a fluorescent microscope (Carl Zeiss Microscopy GmbH, Oberkochen, Germany) to evaluate the viability of bacteria. Bacterial survival was expressed as the number of green bacteria.

### Statistical analysis

The data were analyzed with SPSS (IBM Statistics, Chicago, IL, USA). The Kolmogorov-Smirnov test showed normal distribution of the variables. Independent samples *t* test and paired samples *t* test were used to compare data between different groups. A *P*<0.05 was considered statistically significant.

## Results

No bacterial proliferation was found in the negative control group (Figure 1c). All the positive control samples showed bacterial growth. In both the standard (ProTaper Gold) and experimental (WaveOne) groups, five samples showed bacterial growth in the CFU count test.

**Figure 2:**
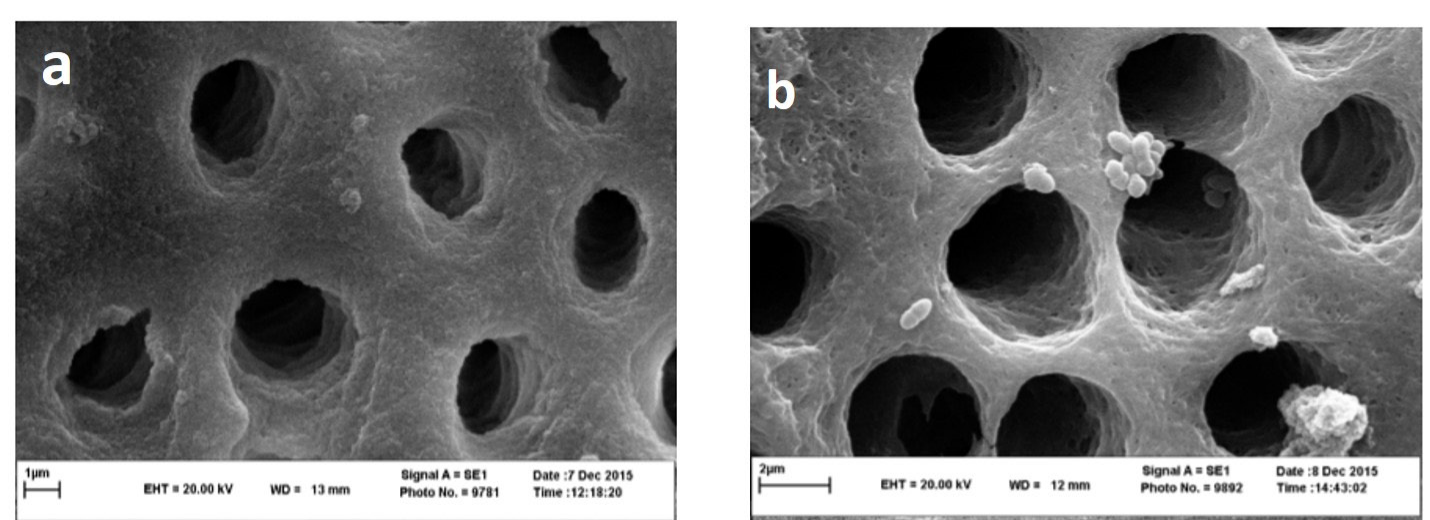
bacterial reduction in WaveOne (a) and ProTaper (b).

SEM evaluation showed a large number of bacteria in the positive control group (Figure 1d), and a reduced number of bacteria in the ProTaper Gold and WaveOne groups (Figures 2a, 2b).

There was a statistically significant difference in the viability of bacteria between ProTaper Gold and WaveOne groups (*P* = 0.004). The viability of bacteria was the highest in the control group, and the ProTaper group exhibited more viable bacteria compared to the WaveOne group (Table 1). There was no statistically significant difference in terms of dead bacteria between the ProTaper Gold and WaveOne groups (*P* = 0.992) (Table 2).

**Table 1:** Means (SD), minimums, and maximums of viable bacterial counts in different experimental groups

**Group**	**Live bacteria**			***P*** ** value**
	**Mean ± SD**	**Min**	**Max**	
ProTaper	2.348±1.100	0.5	4.33	0.004
WaveOne	1.020±0.647	0	2	

**Table 2:** Means (SD), minimums, and maximums of dead bacterial counts in different experimental groups

**Group**	**Dead bacteria**			***P*** ** value**
	**Mean ± SD**	**Min**	**Max**	
ProTaper	0.622±0.346	0.2	1.4	0.992
WaveOne	0.620±0.642	0	1.9	

In the ProTaper Gold group, there was a statistically significant difference in both the dead and viable bacteria (*P* = 0.001); however, in the Wave One group, the difference was not statistically significant (*P* = 0.059) (Table 3).

**Table 3:** Viable and dead bacterial counts in different experimental groups

**Group**	**Bacteria**	**Mean ± SD**	**t**	***P*** ** value**
ProTaper	Live bacteria	2.348±1.100	4.511	0.001
	Dead bacteria	0.622±0.346		
WaveOne	Live Bacteria	1.02±0647	2.162	0.059
	Dead Bacteria	0.620±0.642		

## Discussion

This investigation aimed to evaluate the residing dead and live *E. faecalis* cells following the instrumentation of the root canals with the rotary ProTaper Gold and the WaveOne reciprocating systems. Different studies have shown that mechanical removal through instrumentation mainly contributes to reducing the presence of bacteria and disrupting the bacterial bioﬁlm in the main root canal.^7-10^

In the present study, a mono-species *E. faecalis* biofilm model was selected since it is associated with persistent apical inflammation.^11,12^ It is also able to penetrate the dentinal tubules and escape the chemomechanical disinfection of the root canals.^13^ This in vitro model, introduced by Haapasalo and Orstavik^14^ for the infection of dentinal tubules under controlled conditions, would allow for the efficacy examination of the root canal instrumentation. The maturity of the biofilm also influences its resistance to antimicrobial procedures. Therefore, a 28-day-old biofilm was selected based on previous studies showing that this growth phase can develop a mature biofilm and is optimal in testing the efficacy of disinfection methods.^15,16^

Further examined in the present study were the roots of the premolars. The WaveOne Primary and ProTaper Gold F2 have the same tip size and taper. Studies have shown that root canal preparation with greater size and taper results in greater bacterial count reduction.^17^ However, Machado reported that the use of different tip sizes and tapers resulted in similar bacterial counts.^9^ In this research, for more reliable results, a similar apical preparation was considered for both groups.

In order for the conditions to simulate real clinical conditions, the smear layer was eliminated using 5.25% NaOCl and 17% EDTA before exposing the root canals to *E. faecalis*. Similar to the present study, Coldero et al^18^ and Mohammadzadeh et al^19^ eliminated the smear layer prior to exposing the root canal to the microbial cells.

In the present research, a fluorescent microscope was used for quantifying viable and dead bacteria within dentinal tubules. All the samples in both experimental groups showed live bacterial cells. The number of viable bacteria was significantly low in root canals instrumented with WaveOne, indicating the efficacy of WaveOne in cleaning the canals, and elucidating the significance of the residual bacteria in a nonculturable state. However, there was no significant difference in dead bacteria. In both the ProTaper Gold and WaveOne groups, only 5 samples out of 10 exhibited bacterial growth using the CFU count test. Such discrepancies between the results of the two antimicrobial techniques can be due to the evaluation methodology of the CFU count test, which has no sufficient sensitivity for detecting possible viable cells in lower concentrations.^20^ Furthermore, it has been demonstrated that after facing adverse environmental conditions, many bacteria can enter a viable but nonculturable (VBNC) state.^21^ The clinical importance of this issue is that in VBNC states and when optimal conditions are restored, bacteria are capable of resuming active growth.^22,23^

Bürklein et al^24^ reported that residual debris in root canals was significantly lower in WaveOne instrumentation comparisons with ProTaper instruments. Machado et al^1^ found no signiﬁcant difference in bacterial count reduction among the reciprocating WaveOne system and rotary ProTaper system. Martinho et al^6^ revealed that both WaveOne and ProTaper were similar concerning the effectiveness of decreasing endotoxins and cultivable bacteria from primarily infected root canals. In another study, they showed similar results in endodontic retreatment with WaveOne and ProTaper.^25^ The controversial results of these studies, compared with this study, can be due to our antimicrobial method. The colony count technique was used in the studies mentioned, which is not sensitive, and certain bacteria, although viable, cannot be cultured.

In conclusion, given the limitations of this in vitro study, WaveOne is more effective in eliminating *E. faecalis* from the root canal walls of extracted human teeth.

## Authors’ Contributions

JG designed the study. SM drafted the work. EA designed the study and drafted the work. MG analyzed and interpreted the data. MG drafted the work and prepared the manuscript.

## Acknowledgments

The results presented in this study have been extracted from a student thesis (No. 580) in Mashhad University of Medical Sciences (MUMS).

## Funding

This study was supported by a grant from the Vice Chancellor of Research Council of Mashhad University of Medical Sciences, Iran.

## Competing Interests

The authors declare no competing interests with regards to the authorship and/or publication of this article.

## Ethics Approval

The present in vitro study was approved by the Ethics Committee of Mashhad University of Medical Sciences under the code 31531.
